# Behavioral responses to a cyber attack in a hospital environment

**DOI:** 10.1038/s41598-021-98576-7

**Published:** 2021-09-29

**Authors:** Markus Willing, Christian Dresen, Eva Gerlitz, Maximilian Haering, Matthew Smith, Carmen Binnewies, Tim Guess, Uwe Haverkamp, Sebastian Schinzel

**Affiliations:** 1 University of Muenster, Muenster, Germany; 2 Münster University of Applied Sciences, Muenster, Germany; 3 University of Bonn, Bonn, Germany; 4 University Hospital Münster, Muenster, Germany; 5 Fraunhofer Institute for Communication, Information Processing and Ergonomics, Wachtberg, Germany

**Keywords:** Information technology, Health policy, Medical ethics, Biomedical engineering

## Abstract

Technical and organizational steps are necessary to mitigate cyber threats and reduce risks. Human behavior is the last line of defense for many hospitals and is considered as equally important as technical security. Medical staff must be properly trained to perform such procedures. This paper presents the first qualitative, interdisciplinary research on how members of an intermediate care unit react to a cyberattack against their patient monitoring equipment. We conducted a simulation in a hospital training environment with 20 intensive care nurses. By the end of the experiment, 12 of the 20 participants realized the monitors’ incorrect behavior. We present a qualitative behavior analysis of high performing participants (HPP) and low performing participants (LPP). The HPP showed fewer signs of stress, were easier on their colleagues, and used analog systems more often than the LPP. With 40% of our participants not recognizing the attack, we see room for improvements through the use of proper tools and provision of adequate training to prepare staff for potential attacks in the future.

Critical infrastructure (CI) institutions (e.g., power grids, telecommunication facilities, and healthcare institutions) guarantee the availability of services and goods required by our society and economy^1^. Cyberattacks on CI pose a serious threat as they endanger critical supply chains. Healthcare institutions, especially hospitals, hold a special place among other CI. They serve to maintain public services^2^ and they play a critical role in saving peoples’ lives^3^. Further, they generate, process, and store large amounts of valuable medical data from thousands of patients^4^. However, the combination of poor security of networked medical devices and the surrounding systems^5–11^ as well as few physical entry-barriers make these institutions easy targets for cybercriminals^12–15^. We already encountered large-scale, non-targeted and targeted attacks in the past due to these circumstances^10,16–19^. A study in 2017 reported that up to date, 64% of all German hospitals have become victims of cybercrimes^20^. In addition to non-targeted attacks, hospitals are also targeted by ransomware attackers^19^. The German university medical center of Duesseldorf experienced a large scale ransomware attack in September 2020. This lead to a breakdown of emergency care and major parts of the hospital infrastructure^21^.

Areas of critical patient treatment as operating rooms, emergency departments, intermediate, and intensive care areas are crucial for the patient’s well-being. A system not being available can result in harm or death for patients. Due to the prevalence of networked medical devices, technical attack vectors exist in these systems^22^. Patients depend on the medical equipment working correctly and the medical staff’s correct use of it. In particular, crisis-behaviors play an important role in medical environments^23,24^. Although decisions are made by the medical professional staff, technically supported medical decision-making is essential in critical care areas^23^. Consequently, human behaviors have to be considered when dealing with cyberattacks.

In relation to cyber security matters, the human factor is described either as the “weakest link”^25^ or one of the strongest defenses^24^. Regarding IT-Security incidents in a hospital environment, there are few published studies. Behavioral research on victims of cyberattacks and the consequences of such attacks have applied different approaches to investigate the stress levels^26^, socio-psychological impacts^27^, and effects on team performance in general^28^. These works have highlighted a strong interaction between psychological and technical factors in a subject area of public interest. Given that nurses provide critical care work in a high-demand, low-control^29^, and high-risk environment, their behavior is crucial in ensuring patient safety. Due to time being a critical factor in critical situations, their actions have to be made quickly and under uncertain conditions^30^.

The presented lab study provides insight into the nursing staff’s behaviors during a simulated cyberattack on the monitoring system of an Intermediate Care (IMC) ward. Usually, an IMC Ward consists of advanced monitoring capabilities and the opportunity to apply nearly all intensive care measurements. These facilities are designed for patients who need to be carefully monitored but do not require the full intensive care spectrum^31^. We created a simulation of an attacker manipulating the values of vital monitors to cause false medical decisions. Although this behavior has not yet been observed in reality, it is technically possible (^32^) as long as attackers have access to the hospital network or physical access to the device.

The study is designed to answer the following questions:

1. How do intensive care personnel members react to a cyberattack producing false alarms that could lead to incorrect and dangerous treatment?
2. What are the characteristics of the group of intensive care personnel who are able to recognize that the devices are no longer trustworthy?

We conducted the lab study in a simulation facility of a German hospital. Typical for simulation facilities, the patients were played by actors, and no real medications were given at any time. We also utilized professional look-a-like systems that can be handled just like the real ones.

Within the simulation, the patients received blood thinners and blood pressure stabilizing medication, and the participants were tasked to monitor the patient’s vital signs to ensure that they were being properly medicated. The simulation consisted of five phases: an acclimatization phase in the beginning and four phases in which the attacker took control over a vital monitor one after another. Apart from the cyberattack, the simulation reflected a routine situation of intermediate care nursing staff. Each participant had to provide care for three patients (Pat. 1–3) with support from a Colleague in the Know (CIK) who was played by a professional nurse. The three patients the participants had to take care of were located in separate rooms and were connected to a networked monitoring system, including a central monitoring unit in the hallway. A floor plan can be found in Fig. 1.

**Figure 1:**
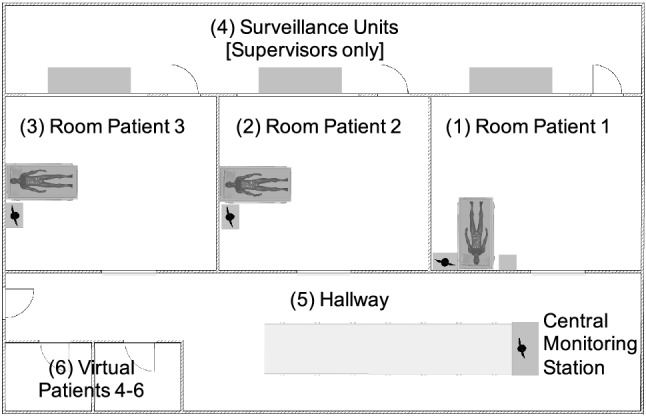
Floor plan of the conducted simulation. (4) was not accessed by the participants.

In the beginning phase (*Acclimatization*) of the simulation, each participant was instructed to perform a routine task to refill the blood-thinning medication of Patient 3, and the CIK left the scene to look after the virtual patients. In each of the next three phases, one new patient’s monitor would sound an alarm and show critical low blood pressure values. However, contradictory to the vital signs displayed on the devices, which suggested the patient’s critical condition, the patients expressed their well-being. In *Phase 4*, all six patient monitors indicated critical low blood pressure levels. In contrast to the monitors, the patients showed neither visual nor verbal conditions that would indicate low blood pressure. The simulation ended on one of two conditions:

- A participant stated that they no longer trusted nor used the values shown on the monitor and stopped trying to stabilize the blood pressure by applying medication or other measures. We classified these participants as (HPP).
- A participant continued to trust the manipulated devices despite the cues given by the CIK in *Phase 4*. Some of them administered a harmful dose of medication due to the misleading readings. If a participant did not clearly reject the displayed values, we classified them as low performing participants (LPP).

As it was crucial for the participants to remain unaware that the study was about their reaction to a cyberattack that had compromised their equipment, the stated goal of the study was to improve workflows. During the debriefing, however, the intention of the study was disclosed, and the participants were reminded of their option to remove their data from the study.

At the same time, as the simulation could cause high-level stress for the participants, psychological support was available. Fortunately, none of the participants had to use this support. The study was completed by 20 participants over the course of six days. Each run was audio and video recorded for subsequent analysis. Our qualitative approach was based on the Co-Act model of Kolbe et al.^33^ which provided us with a framework for observing coordination behavior in acute care teams.

## Results

We present our results in this section. Our analysis is based on the audio and video data recorded during each run. This chapter is structured as follows. First, we present the demographics and general information, such as the simulation lengths and the participants’ categorization. We present data on the participants’ behaviors by focusing on the most prominent expressions and different demeanors in terms of human interactions, sceptical expressions, and emotions. In the end, we compared the high performing participants (HPP) and low performing participants (LPP) groups, categorized the participants’ particular simulation-ending activity, and analyzed the different outcomes.

**Table 1:** Basic demographic information on the participants.

Participants (n = 22)	
High performer	12
Low performer	8
Dropouts	1
Technical problem	1
Sample size	20

Age (Years)	
Range	22 to 51
Average	32.05 ± 7.28

Work experience (Participants)	
1–5 years	4
5–10 years	5
$>10$ years	11

Average phase duration (Minutes)	
Acclimation	3.22 ± 2.01
Phase 1	1.39 ± 0.40
Phase 2	1.26 ± 0.37
Phase 3	1.42 ± 0.36
Phase 4	4.25 ± 2.06

### Overview

A total of 22 participants were recruited. One dropped out, and one data set could not be used due to technical problems during the recording, resulting in a final sample size of 20 participants. Out of these 20 participants, 8 (P1–P8) were placed into the group of low performing participants (LPP) and 12 (P9–P20) were categorized as high performing participants (HPP). The participants’ basic information and demographics are shown in Table 1.

During the *Acclimation*, the participants became familiar with the situation, talked to the patients, and got to know the setting further. In this phase, they were tasked with refilling the blood thinner medication of Patient 3. The duration of this acclimatization phase varied for each participant (see Table 1) and ended when a participant was close to finishing this given routine task. On average, this took about three minutes. In the next three phases, blood pressure alarms went off for individual patients, which were triggered at regular intervals of around 1.5 minutes to create a stressful situation. In *Phase 4*, in which all six patient monitors sounded a critical low blood pressure alarm, the experimenter waited for the participants to show signs of realizing that something was not working well with the patient monitors. The experimenters instructed the Colleague in the Know (CIK) via in-ear communication to give a final cue to the participant before ending the simulation.

None of the participants expressed their lost trust in the devices during *Phases 1–3*, though a few sceptic expressions were made. All participants reached the final *Phase 4*. In *Phase 4*, 12 participants (HPP) already begun to realize that something was wrong with the devices and took measures to protect their patients: *P13:“[You have measured a blood pressure of]. Editorial changes to quotes for a better understanding are provided in square brackets* 120/60? And the alarm is still going? This has to be a technical issue!” All quotes are translated into English from German. Two participants (P11, P15) corrected their incorrect treatment behavior from earlier phases. The remaining eight participants were all categorized as LPP. Four of them stopped all of their actions because of uncertainty and frustration. The four others continued their treatment based on the values shown on the hacked devices and gave harmful treatment to their patients.

**Table 2:**
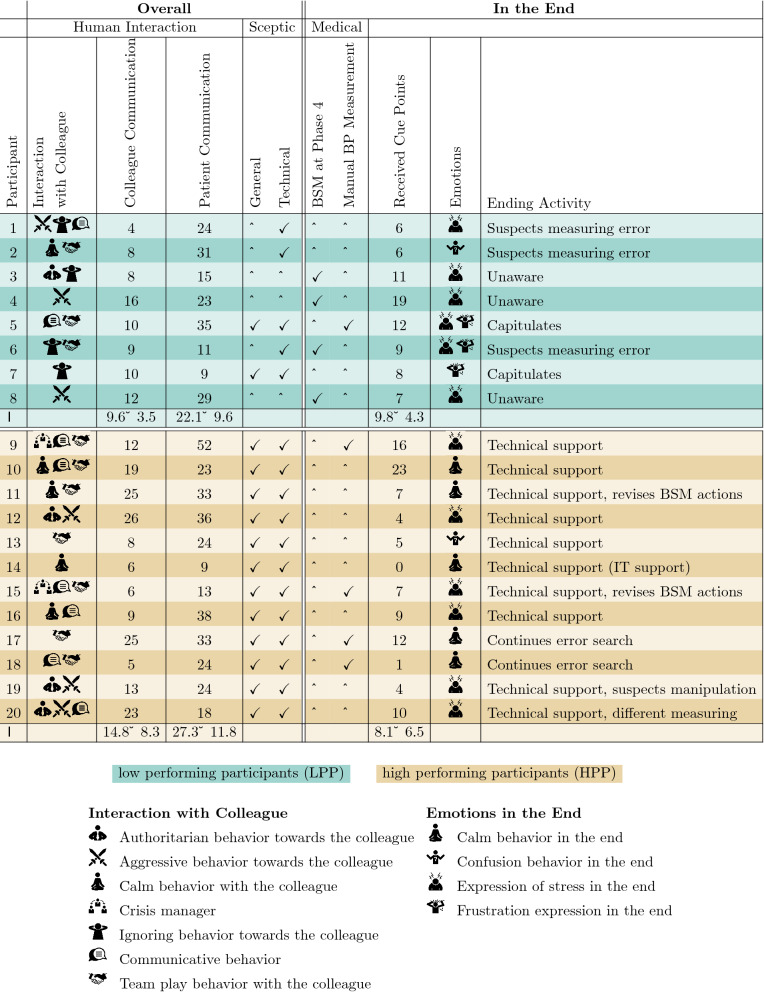
This table presents the participants with their characteristic features in matters of human interaction, scepticism, medical measures, weighted received cue points, emotions, and ending activity.

**Figure 2:**
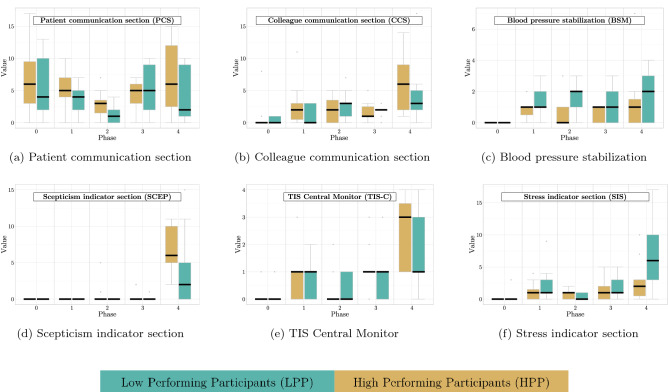
Behavioral patterns of the participants—This figure presents the measured codes, normalized and accumulated into patterns comparing both HPP and LPP through the five phases. Please mind the different scale of Y-axis, Phase 0 was the *Acclimation*.

### Behavioral key factors

**Figure 3:**
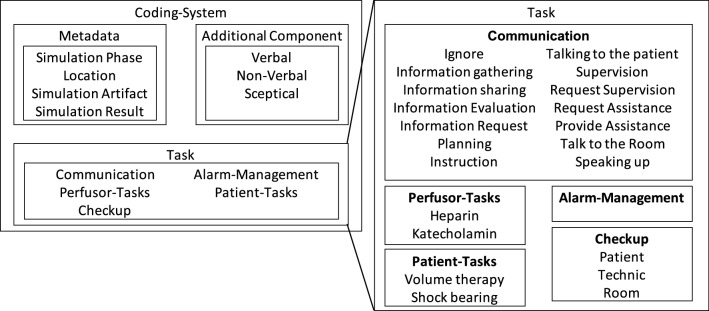
This figure presents the used coding scheme. We modified the established code system of Kolbe et al.^33^ and Burgoon et al.^43^ to cover verbal and non-verbal expressions and combined it with the most prominent tasks of our simulation.

We investigated the behavioral patterns shown by the participants by applying codes describing human interactions, sceptical behaviors, medical measures, and emotional components of behavior on the basis of Kolbe et al.’s Co-Act method^33^ (cf. Fig. 3). An overview of the resulting behavioral factors is provided in Table 2 Further information to Table 2: The participants’ results in this table are summed over the entire course. Thus, superficially contradictory attributes may have been assigned within this integrative image. In the following sections, we present the results based on this table.

#### Human interaction

The observed human interactions included interactions of the participants with the CIK and those with the patients during the whole simulation. Regarding the interactions between the CIK and the participants, we extracted seven categories, which sometimes co-occurred with one another: aggressive behavior, communicative behavior, authoritarian, ignoring, team play, calmness, and crisis manager. We further analyzed the number of interactions with the colleague (CCS) and with the patient (PCS) and compared the groups of HPP and LPP accordingly.

In the following, we present the detailed insights into the participants’ communication behavior based on the two aspects, namely, type of interaction and occurrences.

*Interaction with the colleague* Four participants showed an *authoritarian behavior toward the colleague* (P3, P12, P19 and P20). These participants directed the CIK and assigned tasks either with little consultation or by not asking for their opinion altogether. *(P20 [in an angry tone]:“ Why did you hesitate to come to the patient? [toward the CIK] I do not like that [behavior]! The documentation can wait!”)*. For six participants, we observed *aggressive behavior* toward the CIK (P1, P4, P8, P12, P19, P20). They created a hostile environment by using an aggressive tone and strong language *(P3:“Are you staying here? ... [loudly] are you staying here!?”)*.

In comparison, five participants (P2, P10, P11, P14, and P16) interacted without aggression or stress toward the colleague. They spoke in a relaxed tone and used normal language without expletives. Therefore, they are characterized by *calm behavior with the colleague*. A *Crisis manager* combines team play, communicative behavior, and objectivity and handles the situation ideally (*P15:“Do you have a suggestion on how to proceed...?”[CIK replies negatively] P15:“This definitely qualifies for an emergency; we need reliable monitoring! [CIK agrees] P15 confirms:“Yes, definitely!”*). This characterization was observed for two participants (P9 and P15).

There were four participants (P1, P3, P6, P7) who showed *Ignoring behavior toward the colleague* and did not listen to the CIK. They performed on their own by excluding the CIK from their planning (*After the CIK issued a warning regarding the anomalous situation of all participants having low blood pressure, P3 responded in a snappy tone: “Sometimes that’s just the way it is [sudden drops in blood pressure everywhere]”*. In comparison, nine participants (P1, P5, P9, P10, P15, P16, P17, P18, and P20) were eager to communicate the current situation *(When the CIK pointed toward the central monitoring station, P15 turned toward the CIK and said: “Look at my patients. They all feel fine.”)* thus showing *communicative behavior*. Half of all participants (P2, P5, P6, P9, P10, P11, P13, P15, P18, and P20) showed *team play behavior with the colleague*. These participants offered their help to the CIK, and both, colleague and participant, conducted solution development and local planning activities together: *(P15:“Could you come with me so that we can check whether you are seeing the same value between artery and NIBP. Manual blood pressure measurement (non-invasive blood pressure measurement), is performed regularly on patients in non critical condition because it is easy to complete and has a sufficient resolution. A disadvantage is the duration of minutes until the measurement is complete. Therefore critical patients are often monitored by an arterial blood pressure system, that can detect a critical blood pressure faster and more accurate.*?”

A review of the participants’ behavioral patterns indicates that the amount of interactions (Colleague communication section (CCS)) of the HPP with their colleague in *Phase 4* surpasses that of the LPP in the last phase, shown in Fig. 2b. This matches observations in the literature, wherein colleagues are often identified as an important social support component. Additionally, participants who rely heavily on a team play component also experience greater stress resistance^34–36^.

*Patient communication* The group of LPP had fewer interactions with the patients over the whole simulation period than the HPP (22$\pm 9.6$ vs 27.3$\pm 11.8$). Comparing both groups in *Phase 4* revealed that the median number of PCS of the LPP decreased while the median of the HPP group increased (Fig. 2a) in contrast to the previous phase. The higher number of communication interactions may be an indicator that the HPP lost trust in the displayed vital signs of the monitor and used the patient communication instead as the main control medium to ensure that each patient is stable, whereas the group of LPP focused on blood pressure stabilization, as shown in Fig. 2c. As the patient communication decreased between *Phase 3* and *Phase 4* in the group of LPP, the number of BSM increased (Fig. 2c). This combination indicates that the LPP continued to believe in the shown values. Further, they tended to focus on blood pressure stabilization rather than the expressed well-being of the patients (Fig. 2c).

Our observation of the higher count of interactions (PCS) of the HPP in *Phase 4* matches the literature, in which several studies have identified a correlation between the patient’s outcome and a patient-caregiver communication^37–39^, especially in emergency situations^40^. The same methods that lead to success in the medical setting seem to be applicable to this scenario.

#### Sceptical: general and technical

By reviewing the scepticism expressed by participants concerning the monitor system, we observed four types of participant behavior. These are described below.

1. The group of high performing participants (HPP) succeeded in expressing their scepticism of a general, technical problem and acted accordingly by dismissing the displayed values *(P10:“This has to be a central issue!”, P16:“Maybe it is a technical problem?!”)*.
2. Two members of the low performing participants (LPP) group (P5 and P7) (cf Table 2) also discovered and expressed their scepticism but failed to reject the displayed values. Instead, they decided to stop their activity.
3. Although three LPP participants (P1, P2, and P6) recognized a technical issue with the monitor of one patient, they did not extend this idea to the whole networked system of monitors while surveying the patients on this ward *(P10 toward the CIK after they detached from the displayed values of Pat.2 and Pat.3 :“I believe his [monitoring values of Pat.1] are correct”)*.
4. Three LPP participants (P3, P4, and P8) neither expressed scepticism toward a technical nor a general issue *(P3:“Sometimes that’s [sudden drops in blood pressure everywhere] just the way it is” - CIK:“This does seem a bit strange” - P3:“What do you mean?”)*.

At this point, we analyzed expressions of scepticism per participant in order to review the scepticism in detail. For Fig. 2d, we generated a numerical ranking by applying a factor of five to each forceful expression *(P19:“I am sure we are on the wrong track here; Let us stop here; We are wrong!”)* and a factor of two to each medium expression *(P2:“There is something not working here!”)*. We could not detect prominent scepticism behavior until *Phase 4*. We used the sceptical expressions made when deciding whether participants ended the simulation in a successful manner as mentioned above. As soon as they expressed doubts about the whole monitoring system and stopped trusting the shown values, they were categorized as HPP.

In their work, Ennis et al. identified dispositions and abilities associated with critical thinking: the ability to think in a reasonable manner and the ability to reflect sceptically^41^. Scepticism itself should bring a factor of doubt into consideration, thus allowing one’s current state of knowledge to be incomplete, thereby making other options possible. Evidence has been provided to show that critical thinking ability is not dependable on the person’s personality but on a set of methods aimed at exploring evidence in a particular way^42^, [pp. 1-6].

Further, we reviewed the amount of information gathered by each participant using the central monitoring station (Fig. 2e). Through the central monitor, the participants were able to observe all patient’s blood pressure levels and ECG curves. This central station is separated from the patients’ beds with no direct patient interaction possible.

The data (Fig. 2e) shows that the HPP spent more time in front of the central monitor in *Phase 4* than the LPP, indicating that the HPP group attempted to analyze the whole simulation instead of each patient separately. As this situation required the participants to act against their experience and trained mindset, they had to overcome their desire to treat the patients immediately.

##### Medical: blood pressure stabilization measures in *Phase 4*

 All members of the HPP group ceased performing further medical procedures, such as increasing the Norepinephrine dose or applying volume therapy (e. g. NaCl-infusions to stabilize blood pressure levels) to the patients, once they doubted the displayed blood pressure values. This could be due to the fact that they did not want to endanger the patients by giving wrong medication and/or treatment.

Two participants (P15 and P11) revised previously applied measures (*P15:“Above all, [the patients] receive medication that they may not even need”*).

In the LPP group, four participants (P1, P2, P5, and P7) suspected measurement errors, gave up, and refrained from taking any other actions, including medical procedures and patient communication. Thus they were partially successful within the scenario. However, if this had been a real attack, reactive measures would have been necessary. The other four participants continued with their BSM behavior, as they did not question the displayed values or, as in the case of P6, only suspected a measurement error in one monitor. To review this behavior in detail, we coded and counted all BSM actions and compared groups.

As shown in Table 2, the LPP group used more measures to stabilize the patients’ critical blood pressure levels. We rationalize this difference between the groups in *Phase 4* by assuming that the HPP group already had serious doubts about the correctness of the values. Therefore, they seemed to be more cautious regarding measures to increase blood pressure, especially because this could endanger patients who did not suffer from low blood pressure.

##### Medical: manual measurement

 A manual measurement of blood pressure enabled the participants to detect glaring differences with the displayed values and to discover a monitor malfunction without any trace of doubt. Initially, we hypothesized that this would be a sufficient reason to stop any BSM action. We observed four participants (P9, P15, P17, and P18) in the HPP group and one participant (P5) in the LPP group taking manual measurements. P5 was close to succeeding in the simulation but stopped from further investigating the mismatching monitoring after not finding the error in the first place. P5 was also the strongest patient communicator among the LPP. We had the impression that after they had recognized the mismatch of patient condition and monitoring system, they became so insecure that they did not investigate the issue any further.

#### Received cues

To support the participants and raise awareness regarding the abnormality of the measured values, the CIK was instructed to provide cues. All participants, except P14 (who did not need any), received cues over the course of the study. The CIK was only permitted to provide a limited amount and quality of cues. There were strong cues (CIK toward *P1:“Something may not be correct with the monitoring system...”*), medium cues (CIK towards *P10:“Now it [critical low blood pressure alarm] is happening with all patients”*), and weak cues (CIK towards *P10:“Take a look at this [the central monitoring station].”*). If the participant did not doubt the displayed values in *Phase 4*, the CIK was instructed to give at least one strong cue. Therefore, all LPP received this cue without understanding that the monitors could not be trusted anymore.

To weight the strong and medium cues, we applied a factor of five to every strong cue section and a factor of two to every medium cue section. The group of LPP reached an average of 9.8$\pm 4.3$ cue points and the group of HPP an average of 8.1$\pm 6.5$. In contrast, six HPP (P12, P13, P15, P18, P17, and P19) did not need this final cue at all. The abnormally high value of 23 cue points for P10 (LPP) was caused by their communicative behavior and encouraged the CIK to provide more cues.

#### Emotions

We observed four types of prominent emotions in *Phase 4*: namely stress, confusion, frustration and calmness.

A total of 12 out of 20 participants (P1, P3, P4, P5, P6, P8, P9, P12, P15, P16, P19, and P20) expressed their *stress*, by using expletives, speaking up loudly, or speaking in changed pitch, among others (*P12 [in a grouchy tone]:“Well I don’t know, I can’t take care of 6 patients at the same time...”*). Further, we saw two participants (P2 and P13) expressing their *confusion* in the end (*P2:“That’s very strange indeed.”*). Three participants (P5, P6, and P7) showed *frustration* by expressing how they were incapable of controlling the situation, as none of their actions seemed to have any effect (*P7 [in a huffy tone]:“I wonder what is causing this?!”, “If all patients except one are fine, I don’t know what to do either...”*). Five participants (P1, P3, P6, P17, and P18), all members of the HPP group, showed *calm behavior in the end*, by not showing any strong stress indicators, such as the expressions mentioned above.

After determining stress as the most prominent emotion through the simulation, we reviewed the participants’ stress-related expressions in the same way as their sceptical expressions mentioned above (cf. Fig. 2f). To measure nonverbal behavior as an indicator for stressful behavior, we followed the communication model of Burgoon and Baesler and applied their expression catalog to our data^43^. This model consists of the analysis of macroscopic and microscopic reliability factors of nonverbal expressions.

Both groups (HPP and LPP) follow the same characteristic curve in the Stress indicator section (SIS): it begins with a low base stress level that is increasing in *Phase 1*, decreases in *Phase 2*, rises again in *Phase 3* and reaches its peak in *Phase 4*.

Due to their daily confrontation with stressful situations, members of intensive care staff are considered more stress-tolerant than those who are not working in a similar stressful job^36,44,45^. However, the base stress level is likely to be individually different among the participants and may depend on whether they arrived at the test center after a long shift or directly from their homes.

In the current study, our following interpretation of the observed stress indicators is based on the well-established transactional stress-theory of Richard Lazarus^46^. The model defines three rating phases of a stressful situation: in the primary appraisal, the rating offers three options: The demanding situation can be described as either positive, irrelevant or potentially dangerous (and therefore stressful). In the second appraisal, each participant rates the possible outcome as something that should or should not be managed or (thus making it a stressor). Finally, each participant can react in either a problem-oriented or a emotion-oriented manner^46^.

The first alarm in *Phase 1* introduced a potentially dangerous situation in an unfamiliar setting, leading to the described increased stress levels for both groups. After a short orientation phase, the participants were able to identify the situation as manageable, enabling them to regain subjective control over the situation and act in a problem-oriented way by applying routine procedures to solve the issue. In the next phase, the CIK came and took care of the second patient whose monitor sounded an alarm. The participant can thus rate the situation as irrelevant thereby reducing stress, according to Lazarus. In *Phase 3*, the monitors of all three patients showed critical values. As the CIK was preoccupied with the care of Patient 2, a deficiency situation occured as the participant suddenly needed to care for Patient 1 and Patient 3, who were both in need of treatment. This led to a potentially dangerous situation with an unmanageable outcome that was increased further in *Phase 4*, during which the other three patient monitors (Patient 4–6) are sounded a low blood pressure alarms as well.

#### Endings

In the following section, we present the categorization of all observed endings and a brief appraisal of how the participants are classified. The ending determines whether the participant is classified as LPP or HPP.

##### LPP: unaware

 We observed three participants (P3, P4, and P8) who never doubted the values shown by the patient monitors and, therefore, remained unaware of the cyberattack against their equipment. They continued treating the patients as though the values were correct.

No participant in this group expressed scepticism toward the monitor data (*P3:“Sometimes that’s [sudden drops in blood pressure everywhere] just the way it is”*). These participants also did not double-check the values by measuring the blood pressure manually. All of them continued to treat the patients with BSM until the end of the simulation (*P4:“I’m afraid they’re all critical”*). In matters of human interaction, we observed aggressive behavior toward the colleague from P4 and P8. The other participant (P3) showed authoritarian and ignoring behavior. All unaware LPP participants indicated high stress levels during *Phase 4*.

##### LPP: capitulate

 Two participants (P5 and P7) gave up in *Phase 4*. Both discovered a general and technical issue within the monitoring system (P5 even measured the patient’s blood pressure manually). However, they were unable to interpret the mismatch between the patients’ expressions and the displayed values toward a distinctive decision to detach from the system. Especially P5 made an attempt to find a solution on the basis of issues they might face on a regular basis, but was not able to adapt their mental model to the contradictory information (patient behavior vs. their displayed values) (*P5:“So here’s something wrong with these devices”, CIK:“Yes”, P5:“You know what it is?”, CIK:“No”, P5:“But I know that the cables are all in the right place”[Does not continue to look for other explanations]*). In the end, P5 stopped all of their activities.

Regarding communication, P5 showed good team play and communicative behavior with the CIK, while P7 ignored the CIK altogether. The participants showed stress (P5) and frustration (P5 and P7) in the end (*P7:“If all patients except one are fine, I don’t know what to do either...”* Patient 1 complained about headache and dizziness due to unneeded increased blood pressure medication).

##### LPP: suspects a measurement error

 Three participants (P1, P2, and P6) suspected a measurement error, i.e., incorrect values resulting from wrong calibration or hardware failures but we believe they did not relate this to a defective system.

Even when the CIK gave them cues, one participant (P6) continued to treat the patient with Norepinephrine, which is a potent medicine that is used in emergency situations to rise the blood pressure. Nevertheless, P6 showed signs of good team play throughout the entire simulation and experienced a great deal of stress and frustration in *Phase 4*. P1 behaved aggressively and in an authoritarian manner while interacting with their colleague and ended up showing signs of stress. We observed one confused participant (P2) who showed contradictory behavior. While P2 suspected a technical issue *(P2:“The arterial blood pressure system cannot be trusted anymore!”)* they continued to use the system to measure the blood pressure. This led to confusion as P2 could not understand what was going on (*P2:“If only I knew [what was going on here]”, “That’s very strange indeed!”*). Their confusion seemed to have stopped them from performing additional BSM. P2 stayed calm and showed good team play.

##### HPP: continues error search

 Two participants (P17 and P20) expressed their scepticism, rejected the manipulated values, switched to manual measurement, and adapted their treatment. At this point, they could protect their patients from the cyberattack. However, they tried to find the problem themselves instead of calling the IT-support for help (*P17:“Is the problem with arterial measurement or with the monitors? Or the monitors are off.”*). Toward the end, both participants showed strong team play, communicated well with the CIK, and were very calm.

##### HPP: calling technical support

 We observed 10 participants (P9, P10, P11, P12, P13, P14, P15, P16, P19, and P20) who succeeded in our simulation as they rejected the values and planned to call the technical support for help. This is the ideal reaction, as only technical support can counter the attack in this case. These 10 participants expressed their scepticism about the situation in general and specifically toward the monitoring system ( *P11:“Was there any maintenance or IT related advisory?”, P13:“120/60? And the alarm is still going? This must be a technical issue.”*). All of them stopped their BSM and two also revised previous BSM: (*P11:“Did you adjust the catecholamine dose? I’m also thinking whether we should stop [giving blood pressure medication]... Considering his reaction”. ( As a reaction of Patient 1 to high blood pressure)*”, *P15:“After all, the patients are given medication that they may not even need.”*). Two participants (P9 and P15) checked the blood pressure manually.

In matters of human interaction, we mostly observed positive attributes among the participants, such as strong team play, calm behavior, good communication, and adequate crisis management, yet three participants (P12, P19, and P20) expressed authoritarian and aggressive behaviors. In matters of emotions in *Phase 4*, we observed calmness (P10, P11, P14, P17, and P18), stress (P9, P12, P15, P16, P19, and P20), and confusion (P13). Further, one participant expressed their assumption of data manipulation (*P19:“What do you think? That somebody is messing with us here?”*). One participant (P20) also discussed and planned to establish another measurement method by procuring another monitoring system from other wards.

## Discussion

In the following section, we conclude by discussing our methodology, limitations, results and by reflecting on further research directions.

Our study design examines the behavior of intensive care staff in the face of a cyberattack, which compromised patient monitors by displaying inaccurate values. All participants worked at one of four intensive care wards in the same hospital and all knew that they were part of a study. However, we did not disclose the cyberattack aspect of the study until the debriefing. We also asked them not to disclose the study’s true intent to others. A detailed description of the limitations can be found in the paragraph Limitations in the Methods section.

In all, we observed that 12 of the participants successfully detached from malfunctioning monitoring systems, established an adaptive patient control strategy via communication, and managed the crisis with correct medical procedures. However, eight participants did not attempt to find the correct treatment behavior, thus endangering their patients. This shows an urgent need to raise awareness regarding the dangers of cyberattacks among medical staff and to include such scenarios in training programs. We made several observations that should inform the training programs. First, we noticed that high performing participants (HPP) communicate more with the patients and the Colleague in the Know (CIK) in demanding phases of the simulation. Furthermore, they managed to detach themselves from a single patient treatment toward a view of the entire situation. In comparison, we observed a higher stress level among the low performing participants (LPP). The literature indicates a relation between the patient outcomes and the experienced stress level of the nursing staff^47^. Cooperative team members also seem to have a higher stress resistance then non-cooperative ones^34^. Further, we saw a trend regarding stress and communication behavior. Future studies should investigate whether our results could represent personal healthcare behaviors during cyberattacks or other demanding/ emergency situations. In this context, it should be clarified to what extent the individual disciplines and their personnel differ with regard to their digital attack surface. Specific medical processes and individual practices can make the response to cyberattacks significantly different. In addition, we expect strong differences in terms of the location of the healthcare facility and its specific characteristics such as: Size, capacity, professional diversity, equipment, etc. We consider it especially challenging to achieve meaningful results in this area that are also valid in different departments and across national borders.

In conclusion, personal factors and human behaviors play key roles in reducing risks regarding patient safety, when confronted with cyberattacks. The findings show that proactive communication and the ability to detach from a specific problem are crucial in the successful management of an attack situation. Therefore, increasing awareness and training staff to react properly combined with implementation of supporting guidelines, comprise an effective strategy to cope with related harmful incidents in the future. Based on this study, awareness and training programs can be designed and further studies in broader fields may reveal additional strategies to ensure patient safety in the future.

## Methods

In this section, we describe the methodology and provide insights into our attacker model, the test simulation, the participants, the recruitment process, and the evaluation methods used in this study. All methods were carried out in accordance with relevant guidelines and regulations.

### Participants

The participants (n = 20; 17 females, 3 males, 22–51 years) were recruited via the department of central nursing of the university medical center, which provided us with randomly selected volunteers. The participants were allowed to attend in the study during their work time. Informed consent was obtained from all subjects. No minors participated in our study. We only included nurses in the participant pool if they were active members of the intensive care staff.

In Germany, intensive care nurses are obliged to complete a standardized three-year training and attend an additionally intensive care course. This training prepares them for working in highly technical medical departments, by developing their skills in the regular use of patient monitoring systems, ventilators, dialysis systems, and infusion pumps regularly. Intensive care nurses are required to act independently in emergencies and, for example, are permitted to apply circulatory stabilizing medication such as Norepinephrine^23^. We recruited a total number of 22 participants. One participant withdrew consent, and one video feed was corrupted due to a technical error. Thus, the final data set contained simulation runs from 20 participants.

### Simulation

To create a realistic environment, the simulation replicated a real hospital Intermediate Care (IMC) situation with most of its details during a night shift. We chose a night shift, as it is likely that attackers may wait with their attack until the weekend or the night when only limited personal is available and detection, and mitigation measures may take more time^48^. The ward consisted of six beds in single-bed rooms. Further, it had a central monitoring system for all patients and corresponding individual monitors next to each patient bed. All six beds were occupied by patients. Three of the patients were portrayed by actors, and the other three patients were only shown virtually on the central monitoring system as they were not directly interacting with the participant but created the image of a real intermediate care ward with a regular number of patients. Further, it gave the Colleague in the Know (CIK) a reason to be absent in *Phase 1*, when caring for the virtual patients. The participants were responsible for three patients portrayed by the actors. The CIK supported the participant but was mainly assigned to the three virtual patients. To enforce the authenticity of the simulation, we chose a real nurse to portray the CIK. During our study, the CIK was portrayed by three different actors with the same intensive care training. This way, they were able to perform the interactive communication among themselves and with each participant in a realistic way. While the participants and the CIK worked at the same hospital, they did not know each other prior to the conduct of the study.

Each patient was provided with a name and an individual cover story. The first patient, Patient 1, was an 89-year-old male recovering from a recent heart attack. He was constantly medicated with catecholamine to maintain the stability of his cardiovascular system. An overdose of this medication was dangerous for any of the patients as it could cause a cardio crisis. Patient 1 was especially at risk due to his previous heart attack and was thus considered the most critical patient on the ward. Patient 1 was fully oriented and was able to answer and talk to each participant. His condition was stable, but he was continuously monitored by the ward’s vital sign monitoring system. The second patient, Patient 2, was a 65-year-old male who was being monitored due to his recent unexpected syncope, a single time unconsciousness without any known reasons. He was otherwise in good condition and was able to speak freely to the participants. The third patient, Patient 3, was a 55 years old male who was recovering from a stroke. He was not able to communicate properly with the participants due to the stroke. However, he was fully oriented and medicated with a blood-thinning medication e.g. Heparin. He was monitored accordingly as a precaution. In German wards, men and women are accommodated separately, so all patients were male.

The goal of the simulation was the detection of a general technical issue or even a possible data manipulation with malicious intent. Thus, the simulation ended as soon as a participant states their doubt by rejecting the displayed values, including no longer acting on the basis of this false data and if they suspect manipulation. If this condition was not met, the simulation was terminated by the experimenters.

In the following we present the detailed simulation process: before the simulation began, the participants were introduced to the new environment, their colleague (CIK) and the patients’ backstories. Furthermore, they had the opportunity to ask questions and to get to know the CIK. During this introduction, the participants were instructed to refill the Heparin medication of Patient 3 as it was about to run out. Although the participants were familiar with the task, it still required concentration. This routine task was designed to introduce them to the simulation and to draw their attention to one task. The simulation began with each participant performing an initial checkup for all of their three patients and preparing the Heparin (*Acclimation*). In the meantime, the CIK was occupied with the virtual patients in another room but was available on call. While the participant was busy with refilling the heparin perfusor of Patient 3, *Phase 1* began with the monitoring system of Patient 1 as the attacker’s first target. It sounded an alarm and displayed a critical arterial blood pressure on the monitor in the room as well as on the central monitoring station. The participant had to interrupt the current task and address the alarm while the CIK was preoccupied with their patients and could not assist the participants.

At this point, the participants had several possibilities on how to react to the alarm. Besides talking to the patient who acted and responded as if he was feeling fine, each participant could check the blood pressure with the digital or analog system. However, as the digital system was compromised, it would continue to display wrong values. A stethoscope and a manometer were placed next to the bed of Patient 1 and could be used for manual measurement. As soon as a participant used this manual system, the simulation supervisors told them through a speaker system a physiological blood pressure. This pressure did not match the displayed ones. Besides the information gathering possibilities, the participant could start performing blood pressure stabilization measures (BSM). For example, they could adjust the medication of Patient 1 in order to compensate the decrease of blood pressure by increasing the catecholamine medication. Apart from medication, they could reposition the patient, for example, by applying shock bearing. As the attacker had no access to the real values, none of these actions would change the displayed blood pressure values. However, the actions would increase the real blood pressure of Patient 1. The actors were thus instructed to respond with symptoms of high blood pressure, such as headache and dizziness. Finally, the participant could also call the CIK or their supervising physician to ask for assistance. This physician was only present by telephone and assured the participants of his short arrival. But until then, they had to manage the situation by themselves.

While the participant reacted to the first alarm, the monitoring system of Patient 2 started alarming with a low blood pressure alarm as well, introducing *Phase 2*. The CIK took care of this second alarming patient and informed each participant about this. The participant remained with Patient 1 and continued their treatment. In *Phase 3*, the monitoring system of Patient 3 started alarming. The CIK expressed their occupation with treating Patient 2 and their inability to leave the room. Patient 3 also acted and responded as if he was in a stable condition.

Further progressing in the simulation, *Phase 4* started with the additional alarms of the three virtual patients. This resulted in the CIK informing the participant that now all six patients had low blood pressure, but the three virtual patients are all stable. The CIK started expressing that it is highly unlikely that all patients have low blood pressure at the same time and asks for further instructions. If the participant did not express doubt on the values themselves, the CIK made the assumption that maybe something was going on with the monitoring system and that the presented values could not be trusted anymore. The supervisors ended the simulation shortly after this statement.

After the simulation ended, the participant got some time to calm down and was led into a debriefing room. During the debriefing, the real intention of the simulation was disclosed, and the simulated attack was described in detail.

### Ethics

In order not to influence our participants in advance, it was important not to disclose the intention of the study. In consultation with the Ethics Committee, we thus decided on a concept with a general disclosure without a complete intention description before the simulation and complete disclosure and description during the debriefing. All experimental procedures were reviewed and approved by the Ethics Committee (Ethik-Kommission der Aerztekammer Westfalen-Lippe und der Westfaelischen Wilhelms-Universität Muenster-Studycode:2018-733-f-S). In retrospect, this concept was different from the standard procedure, but the ethics committee and the participants’ feedback was positive and reviewed as appropriate for this kind of research.

### Ideal procedure

Currently, there is no standard operating procedure (SOP) for a cyberattack-situation in a hospital environment. For this reason, we consulted experts in patient safety and trainers for intensive care personnel in order to develop a recommended course of action for this specific simulation.

In the *Acclimation*, the participants should gain an overall picture of the patients and the technology used to monitor and treat them, including a complete checkup and an estimation of the observed intensity of the particular patient. The complete checkup covered functional tests of all medical devices, such as calibration the measuring levels of the arterial blood pressure system, checking peripheral venous catheters, and verifying the alarm limits. During this checkup, the necessity of the first task to change the heparin perfusor of Patient 3 becomes apparent. Therefore, it is recommended to focus and complete this first task.

As the alarm sounds off in *Phase 1*, the participants should complete their current task and then head to the central monitoring station to identify the location of the alarming monitor. Once they arrive in patient room 1, they should start to gather information to gain an overall picture of the situation from both clinical and technical perspectives.

The alarm has to be acknowledged in time, followed by a descriptive communication with the patient and consultation with the colleague. If the colleague is not on-site, they should be called in. All implemented measures for blood pressure stabilization, including catecholamine elevation and shock bearing, should be carried out in a structured manner while monitoring the effectiveness of each measure. At this point of the simulation, the participants should recognize that the measures done are not generating the desired effect on the blood pressure and that the patient expresses feeling of being well in contrast to the displayed values. At this point, the participants are expected to discuss this phenomenon with their colleague and request supervision by the ward physician at an early stage. This request should be formulated with appropriate clarity. By this time, the participants should start the error search by checking the following: Wrong dosage, perfusor defective, wrong syringe clamped. The optimal behavior at this point would be the transition to manual NIBP measurement and to verify of all monitor values.

In *Phase 2*, the participants should discuss what further procedures should be done with their colleague and continue their troubleshooting process. All this time, they should continue to stay on the phone with the physician. The physician should be comprehensively informed about the current status during *Phase 2*. If not already done, the urgency of their assistance should be expressed. The use of keywords like “urgent”, “critical”, “unstable”, “now” is recommended. Troubleshooting should be done in a consistently structured manner. At this point, it would be optimal to perform the transition to manual Non-invasive blood pressure measurement (NIBP) measurement and the questioning of all monitor values.

In *Phase 3*, the participants should recognize the third alarm in time and determine the location and type of the alarm at the central monitor. In this situation, it is important to communicate with the colleague and assess the next steps. On the one hand, the participants can either instruct the CIK to address the newly appeared alarm and go back to Patient 1 themselves. On the other hand, they can inform the CIK about the current situation and the plan to leave Patient 1 and head to Patient 3. The third option of not addressing the new alarm at all would be wrong. Once again, the establishment of a second measurement variant in case of recognizably abstruse values is recommended. At this point, the transition to manual NIBP measurement and questioning all monitor values would be optimal.

In the final Phase the participants should make the decision to interrupt the work and detach themselves from the patients to acquire an overview of the entire situation. A constructive solution development with the colleague is obligatory. Together, they should perform plausibility checks of the monitor values and establish alternative measures to control the patients’ conditions.

### Acting instructions

The following section describes the instructions for the simulation actors.

### Patients

As the simulation took place during a night shift, all patients were asked to act as if they were sleepy during the simulation until each alarm was activated. The corresponding patient awakens slowly, acts confused and is slightly tired, although he feels well otherwise. Patient 3 is no longer able to speak as a result of his recent stroke and can only communicate non-verbally, for example, by hand signals. If the medication is raised during BSM, the actors complain about headaches and dizziness after a short period of time.

### Colleague in the Know (CIK)

The Colleague in the Know (CIK) is a trained nurse who works with the participants during the night shift. The simulation instructors can give instructions to the CIK via in-ear headphones during the entire simulation. They are supposed to work as a team player but should react with ignorance in case of questions to not direct the participant towards a specific technical issue. After a short introduction, the CIK heads over to their patients in a separate room. During the *Acclimation*, the participants are able to call the CIK to ask general questions, for example, how certain technical equipment works. As soon as *Phase 1* begins, the CIK is occupied with their own patients and expresses an inability to assist the participants. In *Phase 2*, the CIK expresses that they will address the second alarm and heads off to see Patient 2. Until *Phase 4*, the CIK remains in the room and responds to requests for assistance by saying they are preoccupied treating Patient 2. If the participants seek help in *Phase 4*, the CIK should passively assist the search for a solution and start giving weak cues. As a last step before ending the simulation, the CIK is instructed to state that there must be something wrong with the medical equipment.

### Attacker model

The simulation represents an everyday life situation of an intensive care nursing staff that escalates step by step. While the nursing staff is used to demanding stressful situations, this study is explicitly designed to be grotesque in the last escalation steps. Such a situation is highly unlikely without external influence. We asked the participants whether they ever experienced a similar situation before. No one did. The attacker has the capabilities to manipulate the monitoring system of the given IMC in any manner. However, they are not able to read any patient data, meaning they cannot adapt the manipulated values to any changes of the patient’s real vital data. In this simulation, the attacker manipulates the blood pressure values on the patient monitors as well as on the central monitoring station. They start by lowering the first three patients’ blood pressure values one after the other until the values suggest a critical patient condition *(Phases 1–3)*. In the last step (*Phase 4*), the attacker lowers the values of the remaining three patients simultaneously. Each new phase starts 1.5 minutes after the previous phase. The attacker can be seen as a technical sophisticated person or organization with or without inside knowledge. The simulation is possible and plausible as an attack, where the attacker proves their ability to modify monitor values of at least one ward, for example to enforce ransom demands. The attacker in this scenario is motivated by causing the most disturbance to enforce, e.g., their ransom demands. It would also be possible for the attacker to mask the attack and avoid detection to get a stronger foothold in the network. Such an attacker should be further investigated and compared to our research attempt. Another possible attack would be a manipulated monitoring device that ignores the patient’s symptoms. In this case, the attack would be masked and more difficult for the nursing staff to detect. The manipulation would be invisible until the patient’s condition becomes critical. Another motivation of our attacker would be to distract IT professionals from the actual attack purposefully. You could furthermore purposefully overwork the staff and wear them down in the long run. In addition, damage to the company’s image would be unavoidable. We evaluate our attack model as more dangerous because you let the staff actively harm the patient. This is done by behavior that they are supposed to use to protect the patient. Indeed, a technical malfunction could theoretically produce a similar error pattern. However, a malfunction that causes an increasing number of identical critical blood pressure events requires a specific accumulation of errors. We rate this as very unlikely but still possible.

### Implementation details

The study was conducted at a specialized training center for medical staff. Facilities like these are not only used for the training of surgical teams and business continuity games, but also for conducting realistic studies within a hospital environment^49,50^.

A pilot study with three participants was performed in advance to validate the designed concept. As a result, several small improvements were introduced, and the concept was adapted towards a more realistic final version. The floor plan of the simulation setup is shown in Fig. 1. As can be seen, it had a professional simulation environment with three individual patient rooms (1–3) and a surveillance unit (4). This unit was hidden to the participants and only used by the experimenters. The patient rooms were connected by a hallway (5), containing the central monitoring station. A small room (6) attached to the hallway was used as a room for the three additional virtual patients. All rooms can be overseen by video and through one-way-mirrors. For the subsequent debriefing, various debriefing rooms were available containing video analysis equipment to analyze the seen behavior together with the participant. The vital signs at the patients’ beds are generated and displayed by the simulation tool SIMStation$^{\copyright }$. This tool can be controlled from the surveillance units and feed several prepared vital signs into the patient’s vital sign monitors. A custom, open-source client-server web application was developed to simulate the central monitoring station in the current study.

Aside from the hospital bed and the vital monitor, each of the three patients played by actors had attached perfusors. The perfusors were ostensibly connected to the patient with a look-a-like cannula. In reality, these perfusors were connected to a hose system collecting the output fluids of the perfusor in a bag under the patient’s bed. Using this technique, the perfusors can run in normal operation mode while the participants can still modify the flow rate. In order to ensure closeness to reality, the patients were connected to ECG and pulse oxymetry, and the participants had to wear their work clothing. A blood pressure cuff and a stethoscope were made available in the room of Patient 1, allowing the participants to re-measure the blood pressure level manually if they had doubts in the values shown by the vital sign monitors. They also dealt intensively with the role assigned to them and showed a high degree of role presence.

### Evaluation methods

The raw data was evaluated and coded using the analytic tool MaxQDA$^{\copyright }$. To analyze the participant’s behavior we used the Co-ACT model of Kolbe et al.^33^ and Judee K. Burgoon^51^ that was specifically designed to analyze team behavior in acute care teams. The coding system is presented in Fig. 3.

Further, we coded every task from its beginning to completion with just one code. In coding specific communication, we coded the period of time in which the entire information was transported, including breaks in the communication. Consequently, each code contained information about the code category, the timing (point of occurrence and duration), and location. In order to avoid potential bias, the second coder repeated 25% of the coding once the first coder had finished, after which the results were compared. Both coders did not exchange their views on the particular runs until the comparison. We reached a Cohen’s Kappa of $\kappa$ = 0.78, representing substantial strength of agreement.

To compare the participants’ behaviors, some of the defined codes were summarized into behavioral patterns. The patterns are presented below.

### Patient communication section (PCS)

The PCS pattern summarizes the codes *Talking to the patient (descriptive and personal*, *Instruction (Patient)*, and *Information gathering (patient)*.

### Colleague communication section (CCS)

The CCS pattern summarizes the codes *Information gathering, Information sharing, Information evaluation, planning, Instruction, Information request, Supervision, Request assistance*, and *Information request*.

### Blood pressure stabilization measures (BSM)

The BSM pattern summarizes the codes *Katecholamin dose increase and bolus*, *Volume therapy*, and *shock bearing*.

### Scepticism indicator section (SCEP)

The SCEP pattern summarizes all sceptical expressions (strong, medium, and small).

### TIS central monitor (TISC)

The TISC pattern summarizes the codes *Monitoring System (Information gathering monitoring system)*.

### Stress indicator section (SIS)

The SIS pattern summarizes all stress-related expressions (strong, medium, and small).

### Limitations

This study has limitations that need to be considered. First, although we introduced a reference for the behaviors of ICU staff during a cyberattack, further studies should investigate other samples to determine the factors influencing the participants’ behaviors during a simulation.

Second, the study was conducted over a six-day period with three different CIK, which may have led to slight variations. Nevertheless, all three had the same instructions, were German certified nurses with at least five years of experience, around 30 years old, and were trained in the university medical center. Thus, we consider this influence as not substantial.

Third, we reviewed the given cues by the CIK and detected no bias injection that would compromise our results. Both groups were given a similar number of cues with a median of seven cues for the HPP and nine for the low performing participants (LPP). Each participant had different stress levels when entering our simulation, as some of them participated in the study right after a shift. As these participants were equally distributed within the HPP and the LPP, we do not consider this factor as biasing. Moreover, all the participants were instructed not to disclose the real study intent to one another.

Although the participants deal with various emergency situations in their daily lives, the simulation in the current study is more of a special nature, as we created an unsolvable situation. We also included many tasks and circumstances taken from their daily work, such as patients, their team-working colleague, the available medical devices, and the working environment. For logistical reasons, we did not include costumes and make-up of the patient actors, which could have made the simulation appear more realistic. Therefore, although the actors were in the patient beds, they had no other similarities with actual patients except for gender. None of the participants expressed any negative impact of this decision in regard to realism. Even though the participants roughly knew how long the simulation would take, no one expressed their idea that the simulation would now be over at any time because the time was up. Some participants even wanted to progress further in the simulation (e.g., some participants did not want to leave their working place, tried to finish the medical procedures, communicate with the patients) after it was already terminated by the experimenters.

Furthermore, our implemented prevailing ratio between nursing staff and patients (1:3) is permissible and common in terms of the legal regulation for night shifts in an intensive care unit. The ratio in intermediate care wards is up to 1:4^52^. Technically and spatially, it was possible for us to reproduce the conditions on a real station close to the original due to the comprehensive equipment of the training facility. The simulation conditions of the *Acclimation to Phase 3* corresponded to situations that may well occur regularly in intensive care units and IMC wards. Furthermore, the participants are used to alarms sounding off regularly. They often have to interrupt their work and ask for help. A lively exchange with colleagues is also essential and common in their daily life.

For this study, we created a playbook for the simulation beforehand to ensure a standardized plot. Therefore, we were able to establish basic similarities between the individual simulation runs, but we also responded to each participant individually. This mainly affects the *Acclimation* and *Phase 4* as shown in Table 1. The time needed to immerse in the simulation in the *Acclimation* was individual for every participant. We addressed this by extending the time until *Phase 1* started. This led to subjective equality of opportunity but also to slight differences comparing all runs. We tried to create a familiar atmosphere by incorporating routines from everyday hospital life, yet it was clear to the participants at all times that it was a simulation. At this point, however, it should be noted that we were positively surprised by the acting performance of the amateur actors. They dealt intensively with the role assigned to them and showed a high degree of role presence.
